# Transformer-based deep learning approach for obstructive sleep apnea detection using single-lead ECG

**DOI:** 10.3389/frai.2026.1727091

**Published:** 2026-02-11

**Authors:** Malak Abdullah Almarshad, Saad Al-Ahmadi, Saiful Islam, Adel Soudani, Ahmed S. BaHammam

**Affiliations:** 1 Computer Science Department, College of Computer and Information Sciences, Imam Mohammad Ibn Saud Islamic University (IMSIU), Riyadh, Saudi Arabia; 2 Computer Science Department, College of Computer and Information Sciences, King Saud University, Riyadh, Saudi Arabia; 3 Department of Computer Engineering, TED University, Ankara, Türkiye; 4 The University Sleep Disorders Center, Department of Medicine, College of Medicine, King Saud University, Riyadh, Saudi Arabia; 5 Strategic Technologies Program of the National Plan for Sciences and Technology and Innovation in the Kingdom of Saudi Arabia, Riyadh, Saudi Arabia

**Keywords:** artificial intelligence (AI), autoscoring, deep learning (DL), electrocardiogram (ECG), healthcare, obstructive sleep apnea (OSA), polysomnography (PSG), time-series classification (TSC)

## Abstract

Obstructive sleep apnea (OSA) results from repeated collapses of the upper airway during sleep, which can lead to serious health complications. Although polysomnography (PSG) is the diagnostic gold standard, it is costly, labor-intensive, and associated with long waiting times. With the rapid evolution of automated scoring solutions and the emergence of machine learning (ML) and deep learning (DL) in many disciplines, there is a need for tools that use fewer signals and can provide accurate diagnoses. DL models can an process large amounts of data and often generalize effectively to new instances. This makes them a suitable choice for classifying continuous time series data. This study introduces a transformer-based deep learning approach using a single-lead electrocardiogram (ECG) for OSA detection. The proposed architecture, designed to handle raw signals with high sampling rates, preserves temporal continuity over unlimited durations. Without any preprocessing, the model tolerates high-noise raw data. The model is tested with different positional embedding techniques. Additionally, a novel positional encoding technique using an autoencoder is introduced. The proposed approach achieves a high F1 score, outperforming other published work by an average margin of more than 13%. In addition, the model classifies apnea episodes at one-second intervals, providing clinicians with nuanced insights.

## 1 Introduction

Obstructive sleep apnea (OSA) is a disorder characterized by repeated partial or complete blockage of the upper airway during sleep (Halani, n.d.). A study in 2019 reported a prevalence of OSA with almost one billion people affected and around 50% prevalence in middle-aged and older adults in some countries (Benjafield et al., 2019). Undiagnosed and untreated OSA can have profound implications, including cardiovascular diseases (CVDs) (Zhao et al., 2024; Cai et al., 2023), stroke, metabolic disease, lower quality of life, and decreased productivity. Given the high prevalence and serious consequences of OSA, greater efforts are required to achieve accurate and early diagnosis (Benjafield et al., 2019).

Polysomnography (PSG) is considered the gold-standard diagnostic test for OSA. PSG is a full or split night study that is done in the sleep unit at the hospital, to monitor patients’ sleep architecture and several respiratory parameters by collecting a bundle of signals. The American Academy of Sleep Medicine (AASM) guidelines for PSG include electroencephalogram (EEG), and chin electromyogram (EMG) for sleep staging, electrocardiogram (ECG) for heart rate (HR) and arrhythmias, thermal sensors and a nasal pressure transducer to monitor respiratory flow, and photoplethysmography (PPG) for oxygen saturation (Almarshad et al., 2022).

**Figure 1:**
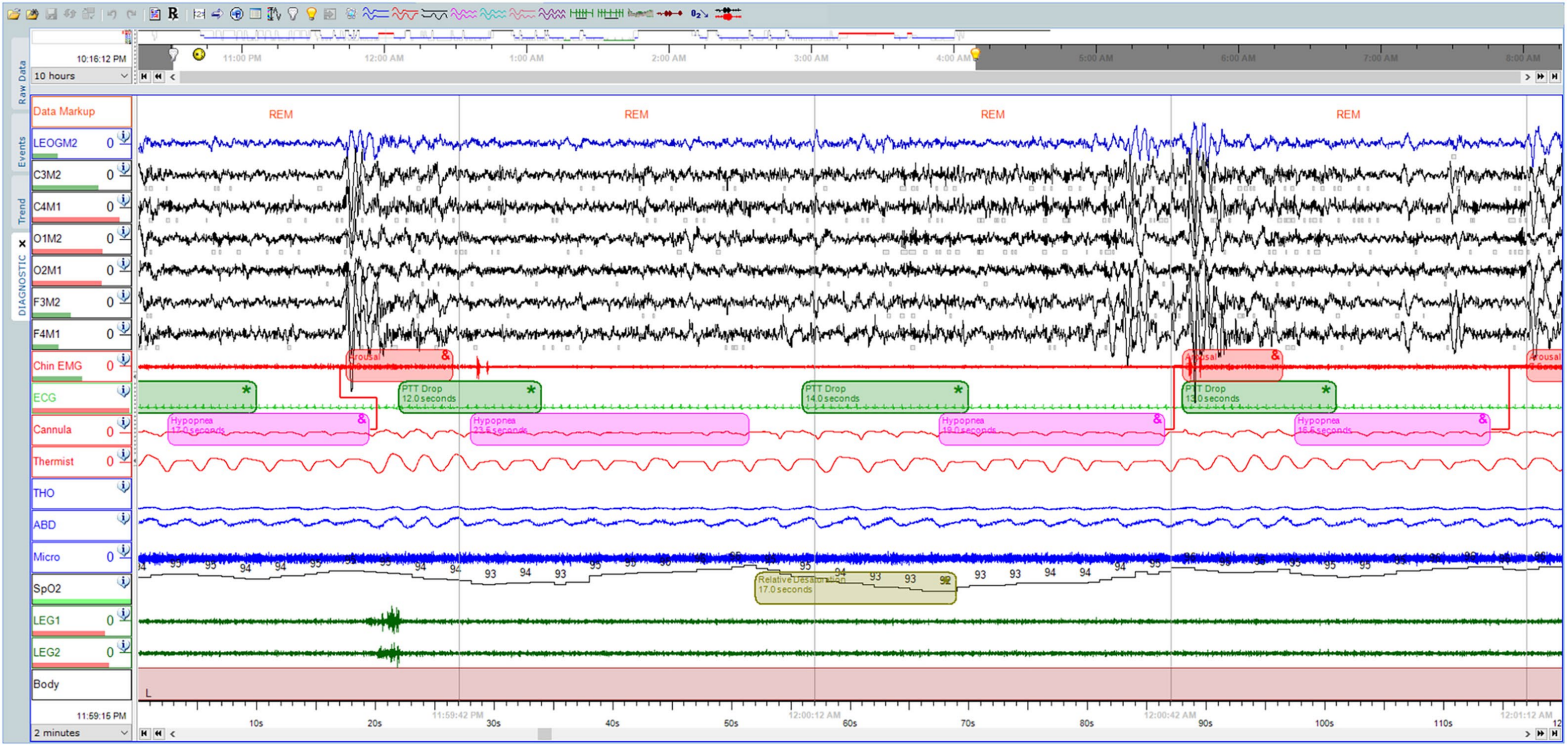
Polysomnographic recording, scored manually by a professional technician at the University Sleep Disorders Center, at King Saud University Medical City (KSUMC), showing some different hypopneas events, within 2 min time window.

Sleep studies (PSG) are expressed as thirty-second epochs of raw data, recorded for 8 h, which are interpreted into around 900 pages (Gupta et al., 2018). Recorded PSG needs a trained technician to score it, which is tedious and time-consuming. To date, the standard PSG scoring process is performed manually (Figure 1). The entire process is complex and costly, potentially causing delays in the diagnosis and treatment of patients with OSA.

Over the last 10 years, various automated scoring solutions have evolved, including statistical analysis, signal processing, machine learning (ML), and deep learning (DL) methods. Several DL network architectures are used for apnea detection, including multilayer perceptron (MLP), convolutional neural networks (CNN), recurrent neural networks (RNN), and long short-term memory (LSTM) (Jahrami et al., 2025). So far, LSTM has achieved the best results (Faust et al., 2021). Although currently available auto-scoring algorithms for sleep apnea have shown promise, there is still a need for further development and validation of these methods that utilize fewer signals and provide more accurate diagnosis.

Among the various signals, ECG signal has a significant role in the detection of OSA as presented in Table 1. Cyclic variations in RR intervals of ECG signals have been reported to correlate with OSA events, resulting in a pattern of bradycardia and tachycardia (Veasey and Rosen, 2019; Mangrum and Dimarco, 2014). Heart rate variability, which can be accurately extracted from the ECG signal, is a key biomarker for the detection of sleep apnea (Verma et al., 2020). This encourages the development of DL models that detect OSA utilizing single-lead ECG exclusively (Table 1). This pattern can be promising in detecting patients with clinical sleep apnea symptoms. However, further research is necessary to validate these findings and assess the reliability of classifying OSA from ECG signals using deep learning.

**Table 1:** Different works for apnea detection from PSG using deep learning, arranged by year of publication.

Paper	Year	Dataset	No. of recordings	Signal type	Classifier	Accuracy
Tagluk and Sezgin (2011)	2011	Proprietary	20	EEG	ANN	96.15%
Guijarro-berdiñas et al. (2012)	2012	Proprietary	6	Nasal airflow and thoracic	ANN	90.27%
Li et al. (2018)	2018	Apnea-ECG Database (Penzel et al., 2000)	70	ECG	DNN	85%
Choi et al. (2018)	2018	Proprietary and MESA (Bild et al., 2002)	179 + 50	Nasal pressure	CNN	96.6%
Urtnasan et al. (2018)	2018	Proprietary	–	ECG	CNN	90–93%
Dey and Chaudhuri (2018)	2018	Apnea-ECG Database (Penzel et al., 2000)	70	ECG	CNN	98.91%
De Falco et al. (2019)	2019	Sleep Heart Health Study	17	ECG	DNN	72.95%
Wang et al. (2019)	2019	Apnea-ECG Database (Penzel et al., 2000)	70	RR	Residual network	94.4%
Arslan et al. (2019)	2019	Proprietary	–	PTT	AlexNet and VGG-16	92.78%
Erdenebayar et al. (2019)	2019	Proprietary	86	ECG	(DNN, 1D CNN, 2D CNN, RNN, LSTM, and GRU)	99.0%
Singh and Majumder (2019)	2019	Apnea-ECG Database (Penzel et al., 2000)	70	ECG	AlexNet	86.22%
Abreu et al. (2020)	2020	Apnea-ECG Database (Penzel et al., 2000) And Proprietary (BrainAnswer RGBT)	–	Respiratory signals	Autoencoder	95 ± 3.5% and 87 ± 6.6%
Urtnasan and Lee (2020)	2020	Proprietary	–	ECG	Deep RNN model utilizes long short-term memory (LSTM) and a gated-recurrent unit (GRU)	98.5–99.0%
Chang et al. (2020)	2020	MIT-BIH Polysomnographic Database (Ichimaru and Moody, 1999) Apnea-ECG Database (Penzel et al., 2000)	–	ECG	One-dimensional (1D) deep convolutional neural network (CNN)	97.1%
Jarchi et al. (2020)	2020	Proprietary	–	ECG and EMG	DNN	72%
Signals (2020)	2020	Proprietary	–	Oronasal thermal airflow (FlowTh), nasal pressure (NPRE), and abdominal respiratory inductance plethysmography (ABD).	Deep BiLSTM-based	85.6
Chang et al. (2020)	2020	Proprietary	–	SpO2 and ECG	LSTMRNN	89.3%
Bernardini et al. (2021)	2021	Apnea-ECG Database (Penzel et al., 2000) and OSASUD (Bernardini et al., 2022)	70 + 30	ECG and SpO2	CNN-LSTM	98.7%
Faust et al. (2021)	2021	MIT-BIH Polysomnographic Database (Ichimaru and Moody, 1999)	–	RR derived from ECG	LSTM	99.80%
Mashrur et al. (2021)	2021	Apnea-ECG Database (Penzel et al., 2000) , UCDDB datasets (Heneghan, 2011)	70	ECG and SpO2	scalogram-based convolutional neural network (SCNN)	95.71
Sheta et al. (2021a)	2021	Apnea-ECG Database (Penzel et al., 2000)	70	RR from ECG	Multiscale dilation attention 1-D convolutional neural network (MSDA-1DCNN) and a weighted-loss time-dependent (WLTD) classification	89.4%
Yue et al. (2021)	2021	2 datasets, their own dataset	–	Nasal pressure airflow signals	multi-resolution residual network (Mr-ResNet)	91.2%,
Zhang et al. (2021)	2021	Apnea-ECG Database (Penzel et al., 2000)	70	ECG	CNN and LSTM	96.1%
Almutairi et al. (2021)	2021	Apnea-ECG Database (Penzel et al., 2000)	70	ECG	CNN-LSTM	94.27%
Drzazga and Cyganek (2021)	2021	SHHS-1 (Quan et al., 1997) and MIT-BIH Polysomnographic Database (Ichimaru and Moody, 1999)	–	Oronasal airflow, the thoracic and abdominal respiratory effort signals.	LSTM	80.66%/82.04%
Leino et al. (2021)	2021	Proprietary	–	SpO2	CNN	88.3%
Sheta et al. (2021b)	2021	Apnea-ECG Database (Penzel et al., 2000)	70	ECG	CNNLSTM	86.25%
Mukherjee et al. (2021)	2021	Apnea-ECG Database (Penzel et al., 2000)	70	ECG	2 CNNs, and CNN + LSTM, MLP based ensemble.	85.58%
Taghizadegan et al. (2021)	2021	UCDDB datasets (Heneghan, 2011)	–	EEG and ECG	an ensemble of recurrence plots (RPs) and pre-trained convolutional neural networks (RPCNNs)	89.45%
Zarei et al. (2022)	2022	UCDDB datasets (Heneghan, 2011)	–	ECG	CNN and LSTM	97.21%
Hu et al. (2022)	2022	Apnea-ECG Database (Penzel et al., 2000)	70	ECG	a hybrid model that contains an altered self-attention mechanism from Transformers	90.5%
Almarshad et al. (2023)	2023	OSASUD (Bernardini et al., 2022)	30	SpO2	Transformers	80.0%
Biswas and Abu Yousuf, (2025)	2024	Apnea-ECG Database (Penzel et al., 2000)	70	ECG	1D CNNs + Transformers	91.97%

Generally, four main architectures of deep networks are used, deep vanilla neural network (DNN) (Li et al., 2018; De Falco et al., 2019; Tagluk and Sezgin, 2011), convolution neural network (CNN) (Dey and Chaudhuri, 2018; Choi et al., 2018; Taghizadegan et al., 2021; Mashrur et al., 2021; Urtnasan et al., 2018), recurrent neural network (RNN) (Signals, 2020; Urtnasan and Lee, 2020) and long short-term memory (LSTM) (Faust et al., 2021; Drzazga and Cyganek, 2021). Some researchers also developed hybrid models (Li et al., 2018; Zhang et al., 2021; Almutairi et al., 2021; Chang et al., 2020; Guijarro-berdiñas et al., 2012; Hu et al., 2022). Shuaicong, et al. developed a hybrid attention model (Hu et al., 2022), utilizing the sinusoidal positional encoding. Recently, Biswas and Abu Yousuf, (2025) achieved state-of-the-art (SOTA) on the apnea-ECG database, using a Transformers based model with a 1D CNNs. Most of these research with coarse-grained apnea labeling (Li et al., 2018; Mostafa et al., 2019; Ismail Fawaz et al., 2019). Additionally, advanced filtering techniques are employed to minimize noise (Bernardini et al., 2022; Bernardini et al., 2021; Sheta et al., 2021b). Furthermore, some approaches depend heavily on extensive data preprocessing and feature extraction (Faust et al., 2021; Sheta et al., 2021a; Wang et al., 2019).

Transformers and their variants, such as BERT, GPT, and ChatGPT, have been proved efficient for multiple natural language processing (NLP) tasks. In this paper, we propose a Transformer-based deep learning framework for classifying PSG as a time series data. An autoencoder with a convolutional transpose (convt) layer is proposed to focus on learning the best representation of the positions of the samples. These samples, along with their learned positional embeddings, were input into Transformer encoder blocks to capture the most relevant information using the self-attention mechanism. Such a model can serve as a component within a larger system to analyze raw signals prior to further processing. Unlike traditional methods that rely on handcrafted features, our approach leverages the Transformer’s self-attention mechanism to automatically capture relevant patterns. In addition, it does not require any preprocessing step, having the ability to deal with raw data of high noise level. Apnea related abnormalities are detected at a one-second granularity, enabling fine-grained temporal localization of clinically marked apnea events. Which provide physicians with detailed insights into the patient’s condition and facilitate the interpretation and validation of the model’s results.

The key contributions of this study can be summarized as follows:

- Developing a Transformer-based model for OSA detection to support clinical decision-making, achieving optimal performance through a novel learnable positional encoding implemented via a simple convolutional autoencoder with a single transposed convolution layer;
- Investigated the impact of various positional embedding strategies on model performance, using static and learnable embedding, and how the proposed learnable embedding using an autoencoder improves the overall model performance;
- Multiple encoding models were proposed for OSA detection; our scheme is the first one that utilizes learnable positioning encoding via an autoencoder and it outperforms all previous models on OSASUD.

The rest of this paper is organized as follows. Section 2 reviews related work and presents different DL approaches to classify apnea. In section 3, we discuss the proposed model and the used dataset. Section 4 explained the experimental setting. Section 5 reports the results in comparison with other deep learning approaches evaluated on the same dataset (Bernardini et al., 2021). Finally, Section 6 concludes the paper and discusses potential directions for future work.

## 2 Related work

Different DL approaches have been proposed in the literature to identify sleep apnea and hypopnea events (Jahrami et al., 2025; Veasey and Rosen, 2019; Mangrum and Dimarco, 2014). We investigated the literature trying to cover published articles in the last two decades. Articles that classify apnea using other techniques, such as statistical methods, signal processing, classical machine learning like support vector machines (SVM), and decision trees (DT), were excluded. Also, papers that deal with other types of apnea detection methods, like processing patients’ videos while sleeping, detecting apnea from wrist actigraphy, smartwatches, or detecting apnea from snoring sounds, are outside the intended context.

Table 1 presents a concise yet comprehensive chronological summary of studies utilizing PSG recordings. For each entry, it provides details on the type of analyzed signal (ECG, SpO₂, or both), the dataset type and name (public or proprietary), population size, signal characteristics, the employed deep learning (DL) model, and its reported accuracy.

Several studies have utilized only one physiological signal to detect apnea events. The vast majority of them used DL on ECG only (Li et al., 2018; De Falco et al., 2019; Dey and Chaudhuri, 2018; Urtnasan et al., 2018; Urtnasan and Lee, 2020; Zhang et al., 2021; Almutairi et al., 2021; Sheta et al., 2021a,b; Erdenebayar et al., 2019; Singh and Majumder, 2019; Zarei et al., 2022; Mukherjee et al., 2021; Chang et al., 2020; Sun et al., 2022); in comparison, some considered SpO2 (Almarshad et al., 2023; Denker, 2022; Leino et al., 2021). Fewer studies took advantage of more than one signal, such as: ECG and SpO2 together (Mashrur et al., 2021; Chang et al., 2020; Bernardini et al., 2021). However, a couple of studies rely on the RR interval derived from ECG (Faust et al., 2021; Sheta et al., 2021a; Wang et al., 2019).

While a few researchers choose to build their own datasets from scratch, most rely on publicly available benchmark datasets. Three datasets are widely used in the literature, namely St. Vincent’s University Hospital/University College Dublin Sleep Apnea Database (Heneghan, 2011), MIT-BIH polysomnographic database (Ichimaru and Moody, 1999), and APNEA-ECG database (Penzel et al., 2000).

Evidence-based medical research depends critically on the availability of raw data of sufficient quantity and quality, not to mention that there are multiple concerns about patients’ privacy, organizational structures, and legal challenges (BaHammam and Chee, 2022). All these contributed to the fact that the most used apnea dataset is two decades old (Chang et al., 2020). Since then, several sleep study standards practices have changed (Padovano et al., 2022). However, multiple papers gained much attention, using their own datasets. Bernardini et al. (2022) published an interesting, comprehensive dataset for 30 admitted patients with precise apnea syndrome severity annotation, and it has been used in their previous work (Bernardini et al., 2021). However, unlike previous datasets, its primary focus is on apnea, and the exclusion criteria were minimal. Consequently, the data are highly susceptible to noise.

## 3 Materials and methods

**Figure 2:**
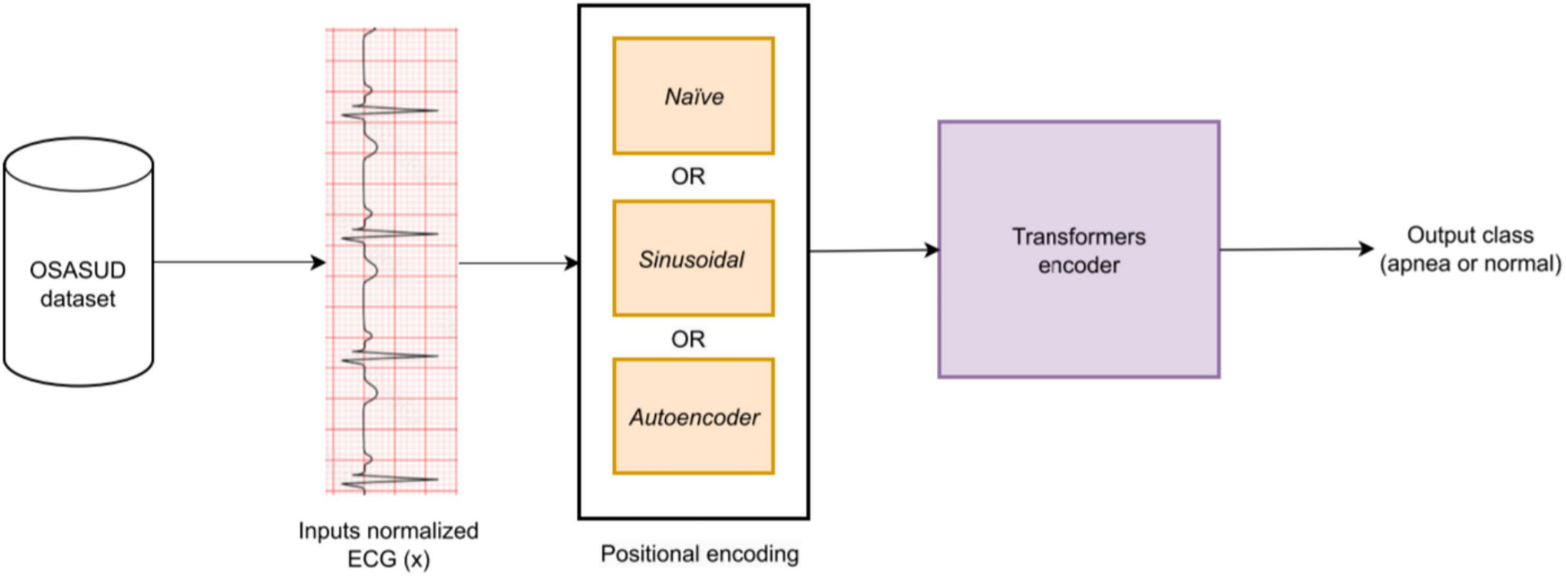
General workflow of the proposed model.

This section presents the dataset, preprocessing steps, and the proposed Transformer-based architecture in a unified and reproducible manner. Figure 2 illustrates the complete processing pipeline, starting from raw ECG input to apnea event classification. The model consists of three main components: (i) input normalization and segmentation, (ii) positional encoding (static or learnable), and (iii) a Transformer encoder followed by a classification head. Our work differs from existing studies in four key ways: (i) it employs a real-world dataset that is both noisy and imbalanced; (ii) apnea events are detected at one-second granularity; (iii) the proposed architecture effectively processes high-frequency raw signals while preserving temporal and spatial dependencies over long durations; and (iv) the model is validated on entirely unseen raw data.

**Figure 3:**
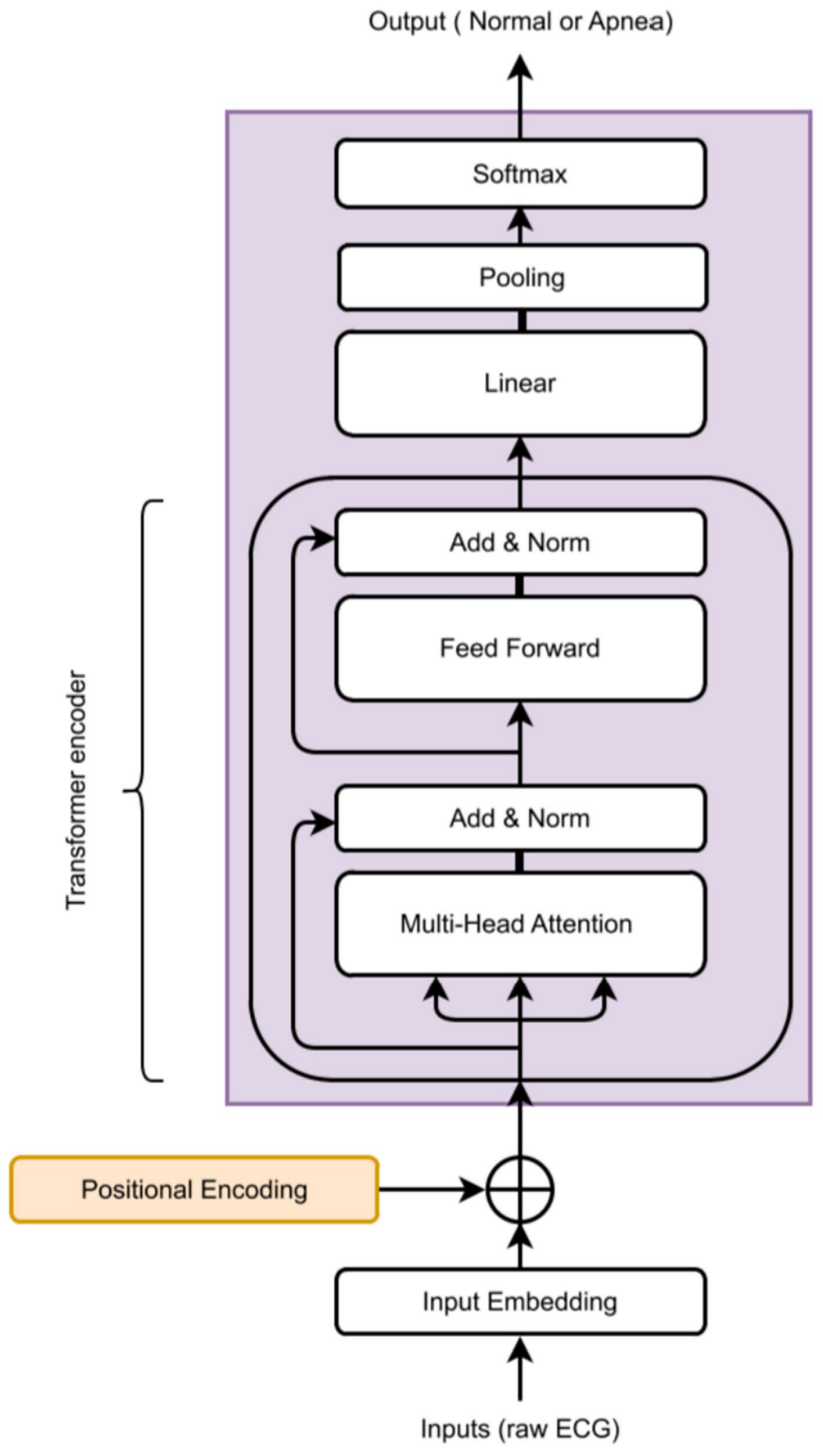
Proposed transformers-base model general architecture.

**Figure 4:**
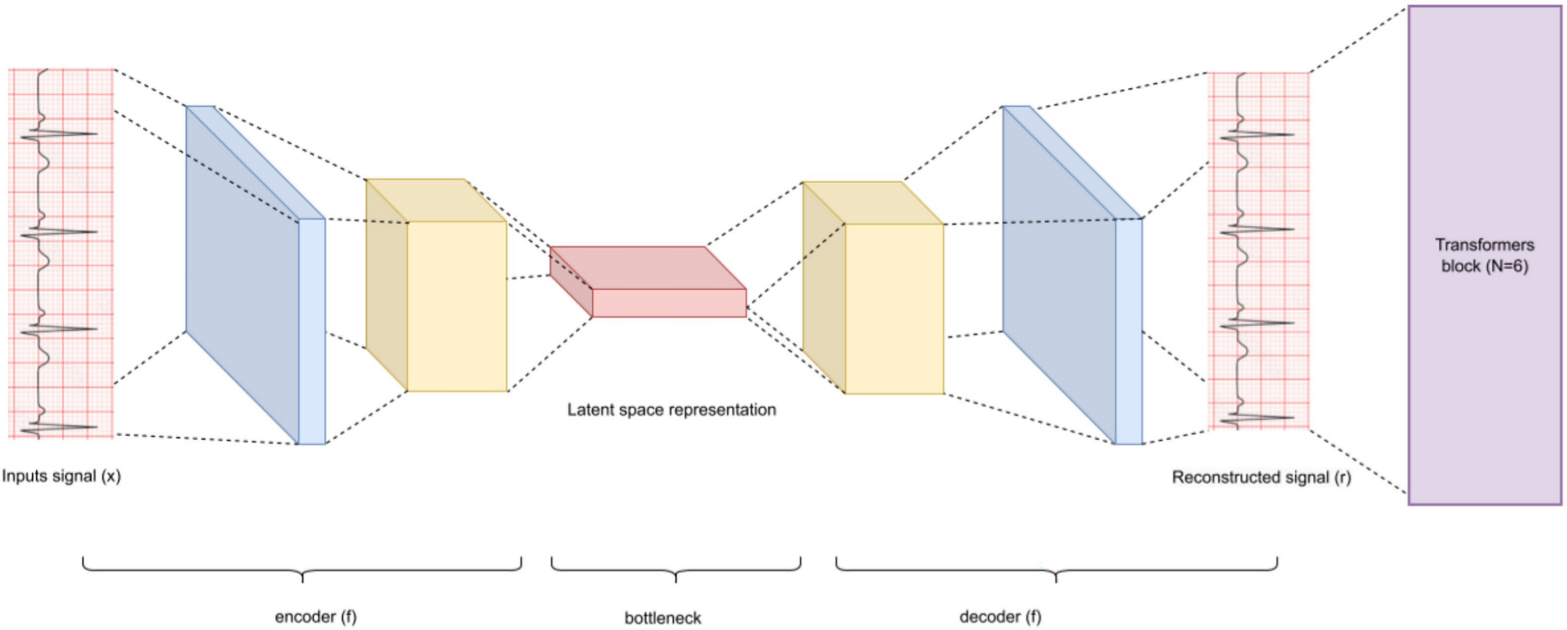
General proposed autoencoder architecture. Autoencoder learns a better representation of each input epoch, then feeds it to the transformer component (Figure 3).

Figure 2 provides a general overview of the proposed architecture. The encoder component is shown in Figures 3, 4 describes the autoencoder positional embedding; The model processes the ECG sampling window as a whole, without shuffling. All experiments were conducted on a local machine equipped with an AMD Ryzen™ 95,900X CPU, an NVIDIA GeForce RTX 3080 GPU, and 32 GB of RAM. The models were developed using TensorFlow 2.10. For reproducibility, the source code of the proposed models are available at: https://github.com/malakalmarshad/TOSA.

### 3.1 Dataset

Most previous studies have been evaluated on idealized datasets, which limits their applicability in real-world scenarios. In contrast, our work utilizes imbalanced and challenging datasets. The dataset we considered is the OSASUD (Bernardini et al., 2022) for detecting OSA syndrome. It consists of 30 patients’ overnight PSG recordings. Patients are affected by different OSAS severity, including 7 subjects without OSA (AHI < 5). Three reasons make OSASUD dataset challenging (Leino et al., 2021); first, PSG (ECG and PPG) recordings are continuous and not segmented into discrete time windows. Second, the dataset is highly contaminated by noise and contains missing and null samples, as is typical in real-world monitoring scenarios. Third, given that the ratio between apnea and normal samples is around 0.17, it is a highly skewed dataset.

A trained sleep technician annotated the collected PSG data for apnea and hypopnea events with one-second temporal resolution. The sampling frequency is 80 Hz for both ECG and PPG. At the same time, high-pass and low-pass filters were set to ECG at 0.3 and 70 Hz, respectively. Each recording has a duration of approximately 7 to 12 h. To train our model, we used ECG lead II (signal_ecg_ii) as on (Bernardini et al., 2021) to compare the obtained result with previous DL models.

### 3.2 Data preprocessing

Trying to comply with the fact that DL models tend to extract features by themselves and require a minimum amount of preprocessing, we preferred not to apply any filters and to assume that our model is capable of dealing with high levels of noise present in the OSUSA dataset. All input arrays were independently rescaled to the range [0, 1] using min-max normalization (Goodfellow et al., 2016). Moreover, all NaN values are replaced by zero. Ground truth anomaly data were segmented into non-overlapping 30-s windows, with each window represented by a list of 30 binary labels indicating the presence (true) or absence (false) of an anomaly at one-second intervals.

In the OSASUD dataset, apnea and hypopnea events are originally annotated by clinicians as continuous temporal intervals, following the standard clinical definition of respiratory events lasting at least 10 s. To obtain one-second level labels, we project each event annotation onto a one-second time grid: all seconds whose timestamps fall within the temporal boundaries of a clinically annotated apnea or hypopnea event are labeled as positive, forming contiguous segments of positive labels that preserve the true event duration. No isolated positive seconds are introduced outside annotated events.

When an apnea event spans across two adjacent 30-s windows, the corresponding seconds in both windows are labeled as positive, thus preserving temporal continuity across window boundaries. The learning objective of the model is second-level classification, aimed at fine-grained temporal detection. However, event-level performance is obtained by aggregating contiguous positive seconds into detected events and comparing them with clinically annotated events, ensuring consistency with the clinical definition of apnea.

### 3.3 Transformers_based model

At the core of our approach for apnea event classification is a Transformer encoder, following the design proposed by Vaswani et al. (2017); We employ only the Transformer encoder, as our aim is to detect apnea events in the ECG signal rather than reconstruct it, a task typically performed by the decoder.

Figure 3 illustrates the generic component of our model, used across all experiments. We refer to Vaswani et al. (2017) for a detailed description of the Transformer, and here highlight our modifications that adapt it for continuous univariate time series classification instead of generating sequences of discrete tokens.

The proposed model takes as input a tensor of shape (batch size, sequence length, 1), where the sequence corresponds to a 30-s ECG window sampled at 80 Hz. Each window is associated with 30 binary labels, representing apnea presence at one-second intervals.

The core of the architecture is a stack of six Transformer encoder blocks, each consisting of a multi-head self-attention layer (4 heads, head size 256) followed by a convolution-based feed-forward sublayer. Residual connections and layer normalization are applied after each sublayer, following the original Transformer design (Vaswani et al., 2017).

To preserve temporal order information, positional embeddings are added to the input sequence prior to the encoder stack. Three positional encoding strategies are evaluated: (i) naive positional encoding, (ii) fixed sinusoidal encoding, and (iii) the proposed learnable encoding based on a convolutional autoencoder. The output of the encoder stack is aggregated using a GlobalAveragePooling1D layer and passed to a multilayer perceptron (MLP) for binary classification.

#### 3.3.1 Encoder stack

The encoder comprises 6 identical layers, each with two sub-layers. The first sub-layer is a multi-head self-attention mechanism with 4 heads of size 256. The second sub-layer uses two convolutions with a ReLU activation in between. Following the original Transformer design, each sub-layer is enclosed by a residual connection and a subsequent normalization layer.

The core part of our model is now completed. Multiple of those Transformer encoder blocks can be piled, but the best results we achieved empirically is by stacking 6 of them. A random search was obtained to find optimized combinations of hyperparameters to train the model. These hyperparameters include the number of heads and the number of encoder blocks (Table 2). To compress the output tensor of the encoder, a GlobalAveragePooling1D layer is added before the final MLP classification head.

**Table 2:** Hyperparameter tuning using random search.

Name	Tested	Best
Batch size	4, 8, 16, 32, 64, more than 64 generates OOM error	32
Optimizer	RMSprop, Adam, Adam(amsgrad), AdamW	AdamW
Initial learning rate	1e-2, 1e-3, 1e-4, 1e-5, 1e-6	1e-3
Learning rate scheduler	Constant, Learning rate decay	1e-4
head_size	128, 256	256
num_heads	4, 8	4
num_transformer_blocks	4, 6, 8, 12	6
mlp_units	258, 128	128
dropout = 0.1	None, 0.1, 0.5	0.1

Alongside multi-head attention, positional embeddings allow Transformers to outperform earlier architectures. Without recurrence or convolution, positional encodings added to input embeddings at the base of the encoder and decoder stacks preserve positional information throughout all Transformer blocks (Vaswani et al., 2017). Sequence order is critical in time series data. Unlike CNNs, RNNs, and LSTMs, which inherently capture order, Transformers replace recurrence with multi-head self-attention for faster, parallelized training. We evaluated three positional embedding strategies: naive, fixed sinusoidal, and learned embeddings (Wang and Chen, 2020; Wang et al., 2022).

Without positional encoding, samples are treated as a bag of words. Positional embeddings are directly added to the sequence representation as (Equation 1):


(1) $$Z_{i=}\text{input}E_{(x_{i})+}PE_{\left(i\right)}$$

Here, $x_{i}$ is the sequence at the *i-th* position, *inputE* the input embedding, and *PE* the positional encoding, which may be learnable or predefined.

#### 3.3.2 Naive positional encoding

It iss a finite-dimensional representation of each sample’s index in a sequence. For a sequence, X = [ $x_{0}$ , …, $x_{k}$ , $x_{n-1}$ , $x_{n}$ ], the encoding tensor informs the model of the position of each element $x_{i}$ is in the sequence *X*. A fixed positional encoding can be calculated from the normalized sequence index as follows (Equation 2):


(2) $$\mathrm{PE}\;(i)=\frac{\mathrm{pos}(x_{i})-\min(\mathrm{pos}(x))}{\max(\mathrm{pos}(x))-\min(\mathrm{pos}(x))}$$

Where *pos* is the position and *i* is the dimension.

#### 3.3.3 Sinusoidal positional encoding

Sinusoidal positional encoding employs sine and cosine functions of different frequencies to represent each sequence position as a vector of size *^d^_model_* (Equation 3):


$$PE_{\left(\mathrm{pos},2i\right)}=\sin\;(\mathrm{pos}/{10000^{2i/d}}_{\text{model}})$$

(3) $$PE_{\left(\mathrm{pos},2i+1\right)}=\cos\;(\mathrm{pos}/{10000^{2i/d}}_{\text{model}})\;$$

**Figure 5:**
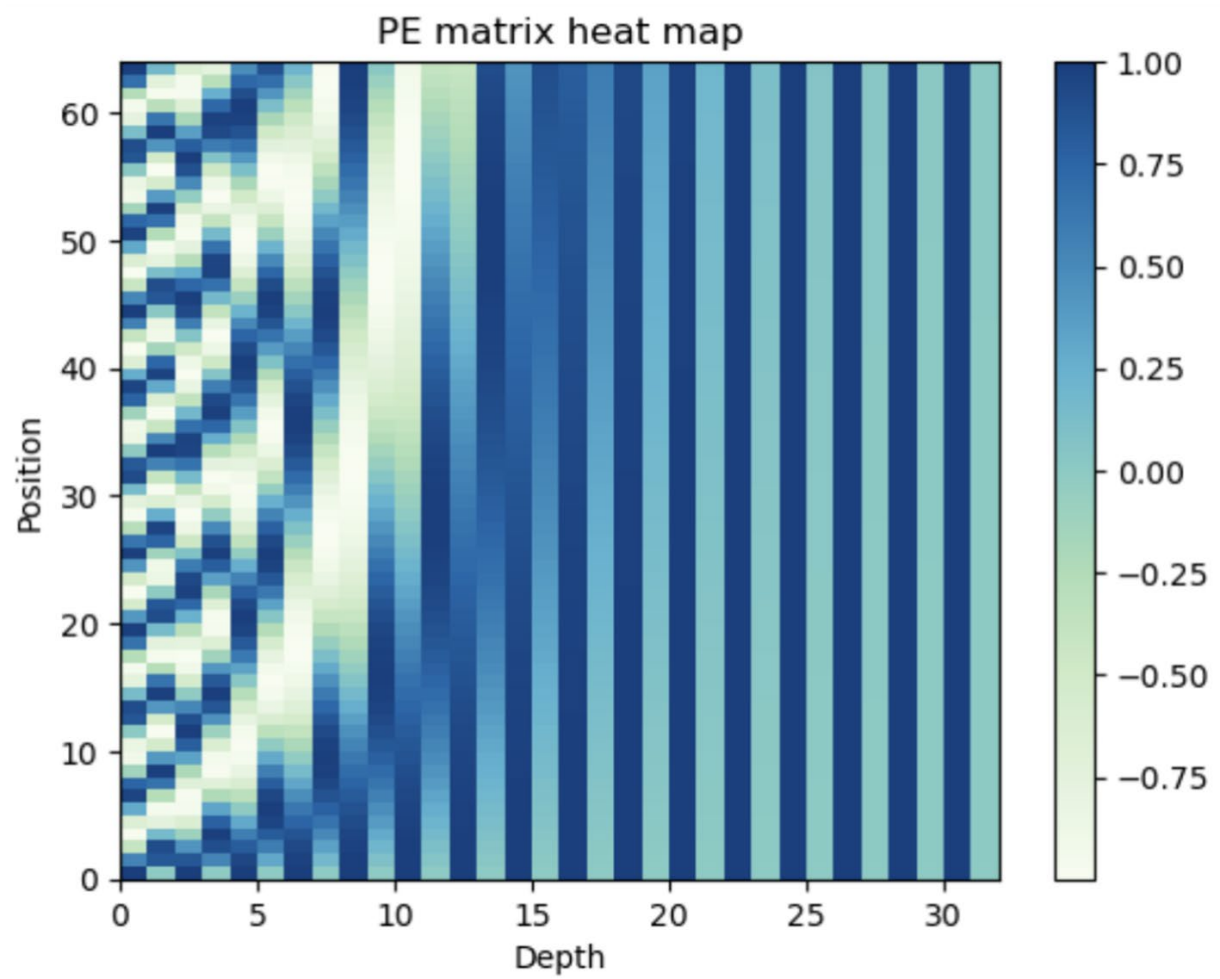
The 64-dimensional positional encoding for a sequence with a maximum depth (dmodel) of 32. Each row represents the embedding vector.

Where *pos* is the position and *i* is the dimension. *2i* and *2i + 1* are used to alternate between even and odd sequences. We experimented with different lengths and depths of the sinusoidal embedding. A length of 64 and a depth (*d_model_*) of 32 provided the most suitable configuration for the dataset. Figure 5, depicts the PE vectors (Vaswani et al., 2017).

### 3.4 Learned positional embedding

Positional encoding is a technique that incorporates information about the position of each token in the sequence to the input embeddings (Vaswani et al., 2017). This allows Transformers to understand the relative or absolute position of tokens which is important for differentiating between events in different positions and capturing the structure of a segment. Standard positional encoding techniques in Transformers can be broadly categorized into absolute encodings and relative encodings that model pairwise position differences. These methods assign positional information solely based on the index of a token in the sequence, independently of the input signal itself. In contrast, the proposed autoencoder-based positional representation is a data-driven, learnable positional encoding. Instead of encoding position as a function of the time index alone, the autoencoder learns a position-dependent representation directly from the raw ECG waveform. Therefore, the proposed method provides a content-aware positional embedding.

The autoencoder consists of two one-dimensional convolutional layers with ReLU activation (filters: 132 and 64), followed by a dropout layer (rate = 0.1), and a transposed convolution layer used for upsampling and reconstruction. The latent representation learned by the encoder serves as a positional embedding that is added to the input signal before entering the Transformer encoder blocks (Figure 4).

This design enables the model to capture both local temporal structure and global positional context, improving robustness to noise and signal variability commonly observed in real-world PSG recordings.

Autoencoders come in handy for data denoising, reducing dimensionality, or even learning a better representation of the samples’ distribution. The critical task was to tweak an autoencoder that fits the job by determining how many layers, different filters are in those layers, and what is the size of the kernel, where kernel size defines the size of the sliding window.

In this work, we employ two 1D convolution kernels with ReLU activation and a filter size of 132 and 64, respectively, and a dropout of 0.1 between them. Then a transposed convolution (convt) as the final layer of the autoencoder. Transposed convolution (convt) reverses the standard convolution by dimensions and is usually carried out for upsampling. Convt can be defined to increase the spatial resolution of feature maps in AE (Dumoulin and Visin, 2016). We choose convolutional autoencoders over feedforward autoencoders, as the convolution layer is better for capturing spatial information (Zhang, 2015).

## 4 Experimental setting

Guided by Occam’s Razor, we started with a simple design and gradually increased complexity, conducting experiments that produced a model performing effectively on a real-world dataset of approximately 1 million samples. More specifically, we started the hyperparameter tuning through a random search through K-fold cross-validation on the training samples. Cross-validation (CV) is a technique for evaluating machine learning models. In k-fold CV, the dataset is split into k roughly equal folds, with one fold used for validation and the remaining k-1 folds for training. This process is repeated k times, reducing variance by utilizing the entire dataset for both training and validation. Nevertheless, it requires a higher computational cost and takes more time because the model needs to be trained K times at the validation step and an additional one at the test phase. In the experiment, we did k = 5 CV.

The dataset was first split at the patient level into two disjoint subsets: 80% of the patients were used for model development and 20% were held out as an independent test set for final evaluation. Within the training subset, we performed 5-fold cross-validation at the patient level, such that in each fold, data from 23 patients were used for training and data from 7 patients were used for validation, with no patient appearing in both sets. This protocol prevents data leakage across folds and ensures that all reported results are generalized to unseen subjects.

We conducted two sets of experiments: first, evaluating the impact of three different positional encoding strategies, and second, investigating training acceleration through weight decay (Loshchilov and Hutter, 2019).

The first set of experiments looks at the effect of different positional embeddings with the base Transformers model on the apnea classification task. To overcome the skewed distribution of the classes in this dataset, different weights were assigned to both the majority and minority classes; this influenced the training to be fair for both classes.

Let TP denote samples correctly identified as apnea, and TN denote samples correctly identified as normal. The evaluation metrics computed from the confusion matrix (Table 3) include accuracy, recall (sensitivity), specificity, and F1-score.

**Table 3:** Confusion matrix.

PR/GT	Ground truth (GT)	
Predicted (PR)	True positive (TP)	False positive (FP)
	False negative (FN)	True negative (TN)

Accuracy: measures the proportion of correct predictions made by the model.

Since the used dataset is imbalanced, which is common in problems of similar nature, where there are fewer anomalies than normal events, accuracy alone is not sufficient to correctly evaluate the model’s performance. To overcome its limitations, we also took into account recall (Sensitivity), specificity and.

F1-Score. We report accuracy, sensitivity (recall), specificity, precision, and F1-score using their standard definitions commonly adopted in the literature.

In addition, we considered the area under the receiver operating characteristic (ROC) curve as a complementary evaluation metric, which shows TP rates against FP rate, illustrating the model’s ability to distinguish between the two classes, whereas a random classifier would not exceed an AUC of 0.5.

It is worth noting that we were able to speed up the model convergence 0.3x faster, while achieving comparable performance, using AdamW with weight decay equal to 0.0001 (Loshchilov and Hutter, 2019).

## 5 Results and discussion

We conducted five experiments using a slightly modified version of the model each time. All experiments were conducted on the OSASUD dataset (Bernardini et al., 2022), with results summarized in Table 4. Initially, we used only the encoder component of the Transformer without any positional embedding (model_1) and employed the AMSGrad variant of the Adam optimizer.

**Table 4:** Performance on the OSASUD dataset.

No.	Model	Accuracy	Sensitivity	Specificity	F1	AUC
1	ResNet (all patients) (Bernardini et al., 2021)	0.716	0.168	0.824	0.162	0.523
2	LSTM + CNN (all patients) (Bernardini et al., 2021)	0.769	0.628	0.769	0.471	0.750
3	ResNet (without validation patients) (Bernardini et al., 2021)	0.737	0.107	0.865	0.737	0.525
4	LSTM + CNN (without validation patients) (Bernardini et al., 2021)	0.752	0.643	0.752	0.468	0.760
5	Transformers (encoder + Adam) (amsgrad = True) (model_1)	**0.7754**	0.8903	0.8750	**0.8638**	0.7928
6	Naïve positional embedding encoder (model_2)	0.7278	0.8909	0.8590	0.8286	0.7644
7	Sinusoidal positional embeddings Transformer (model_3)	0.7428	0.8884	0.8670	0.8404	0.7801
8	Transformers (encoder + weight decay) (model_4)	0.7736	0.8848	0.9075	0.8635	0.8276
9	Transformers (with autoencoder) (model_5)	0.7745	**0.9058**	**0.9277**	0.8606	**0.8520**

The bold values represent the maximum value for each metric.

In the second and third experiments (model_2 and model_3), the sample order for each batch was incorporated using the naive and sinusoidal positional encoding strategies, respectively. These two fixed positional encodings did not noticeably improve the model’s overall performance. Nevertheless, we further explored different parameter values for the sinusoidal positional encoding. After that, in the fourth experiment (model_4), we tried to speed up the training process through the use of weight decay, and it produced similar results and converged about 0.3 times faster with slightly better specificity and AUC.

**Figure 6:**
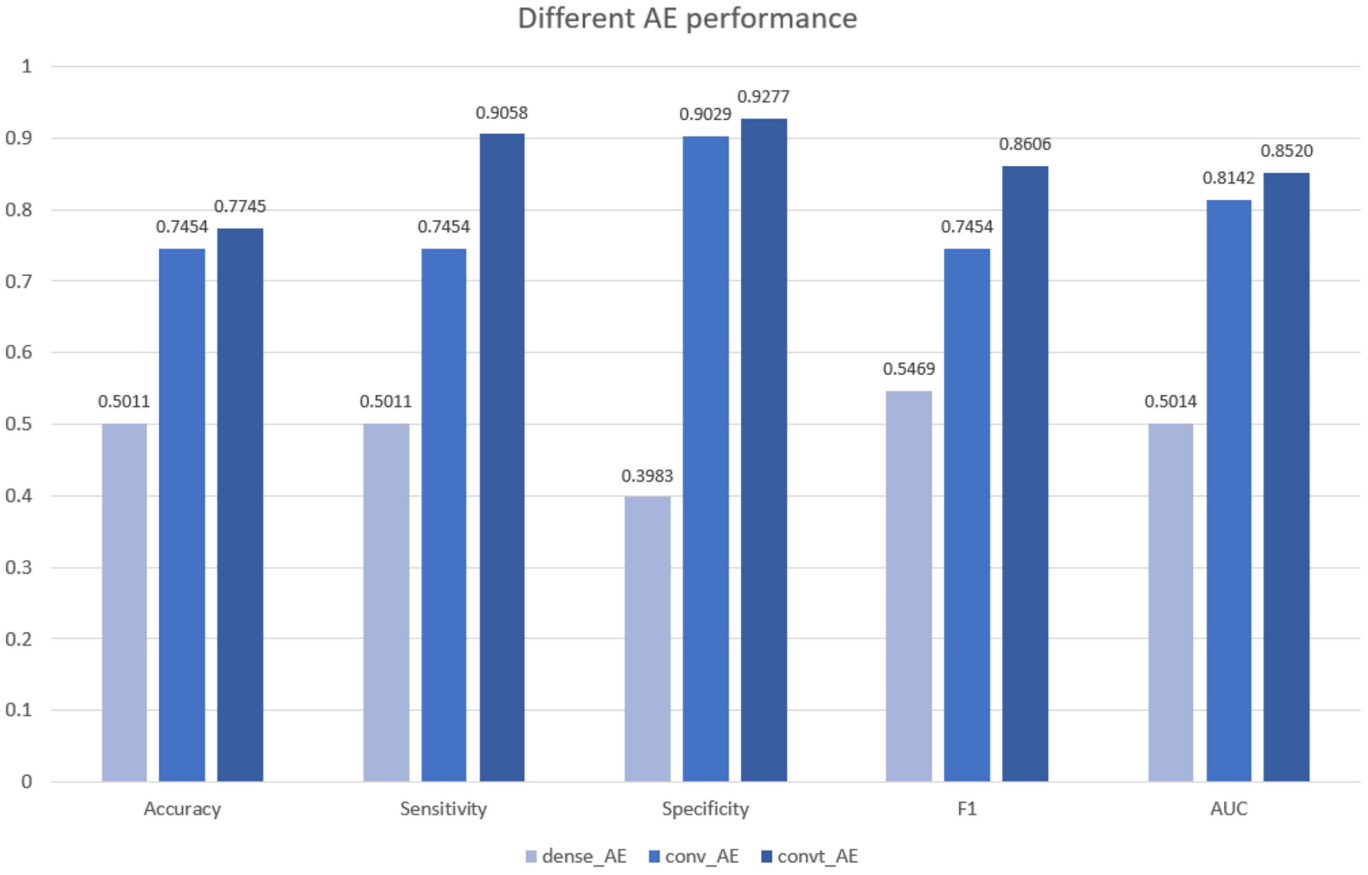
Performance comparison of different AEs as a positional encoding component, AE with a convt layer achieved the best results.

Autoencoders have attracted so much research attention; they have long been thought to be a potential avenue for cracking unsupervised learning problems, i.e., learning useful representations without labels. To be more precise, autoencoders are a self-supervised technique, where the targets are generated from the input. To get self-supervised models to learn features, you have to come up with the right combination of autoencoder layers. To achieve that, we started with the simplest autoencoder based on fully-connected layers (Goodfellow et al., 2016), which predicted approximately similar to a random classifier (AUC = 0.501), and classifies all apnea events as normal events (the majority class). After that, we attempted a convolution autoencoder, that contained only 1-D convolution layers. At this phase, the model shows better separability reaching AUC equal to 0.814. Finally, after adding a transposed convolution layer (model_5) the model achieved its best performance, reaching AUC = 0.852 (Figure 6). The superiority of convolutional autoencoder over dense autoencoder was expected; Convolutional shows exceptional performance before on time series encoding, i.e., Rocket (Dempster et al., 2020).

In the final experiment (model_5), the static positional encoding was replaced with a learnable positional encoding implemented through a convolutional autoencoder containing a transposed convolution (ConvT) layer, added prior to the encoder components. As shown in Table 4, this configuration achieved the highest performance across all evaluation metrics. It is important to consider the implications of emphasizing different metrics. In medical applications for disease prediction, sensitivity is particularly critical, as it is generally preferable to classify a healthy individual as diseased rather than to misclassify a diseased individual as healthy. Notably, model_5 achieved the highest sensitivity among all evaluated models.

Transformer-based approaches have recently been applied to obstructive sleep apnea detection from ECG signals. For instance, Biswas and Abu Yousuf, (2025) proposed a CNN–Transformer model using static positional encoding and segmented inputs. While effective, such designs rely on predefined positional representations and may be less robust when applied to continuous, noisy clinical recordings.

In contrast, the proposed framework employs a learnable positional encoding based on a convolutional autoencoder, enabling temporal position information to be inferred directly from raw ECG signals. By avoiding handcrafted feature extraction and operating at a one-second temporal resolution, the model better adapts to real-world data variability and demonstrates improved generalization on the imbalanced OSASUD dataset (Bernardini et al., 2022).

When it comes to selecting a dataset that is better suited for Transformer-based models, the Apnea-ECG dataset is useful for benchmarking and consists of continuous single-lead ECG overnight recordings lasting approximately 7–10 h, with apnea annotations at one-minute resolution. OSASUD is a more challenging, realistic, and clinically relevant dataset, with apnea annotations at one-minute resolution. Moreover, OSASUD provides a higher sampling rate, which better exploits the strengths of Transformer architectures in modeling fine-grained temporal dependencies and long-range relationships in dense time-series data.

**Figure 7:**
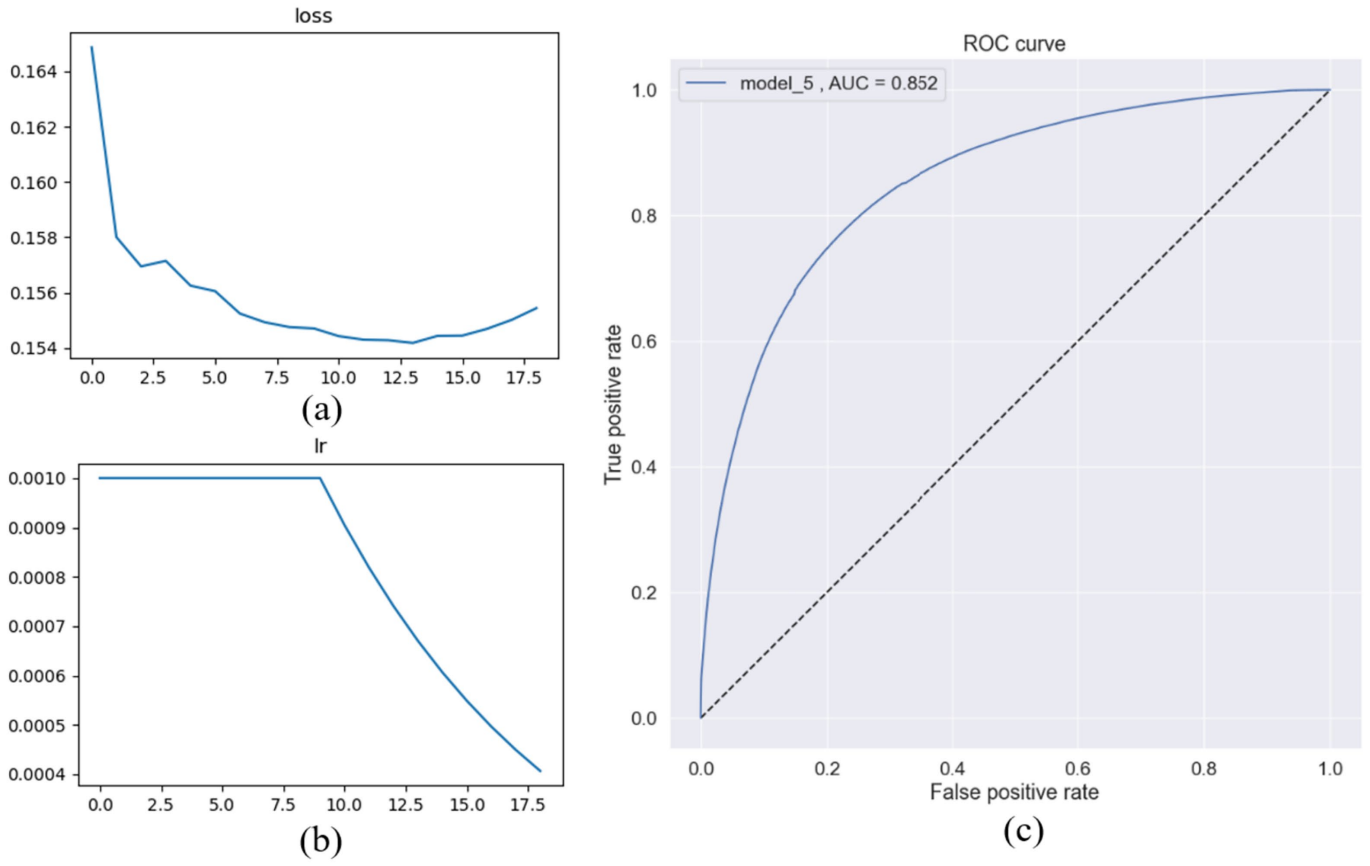
Transformer-based architecture after adding the autoencoder component (model_5): **(a)** Training loss, **(b)** learning rate decreased exponentially after the 10th epoch, **(c)** ROC curve.

**Figure 8:**
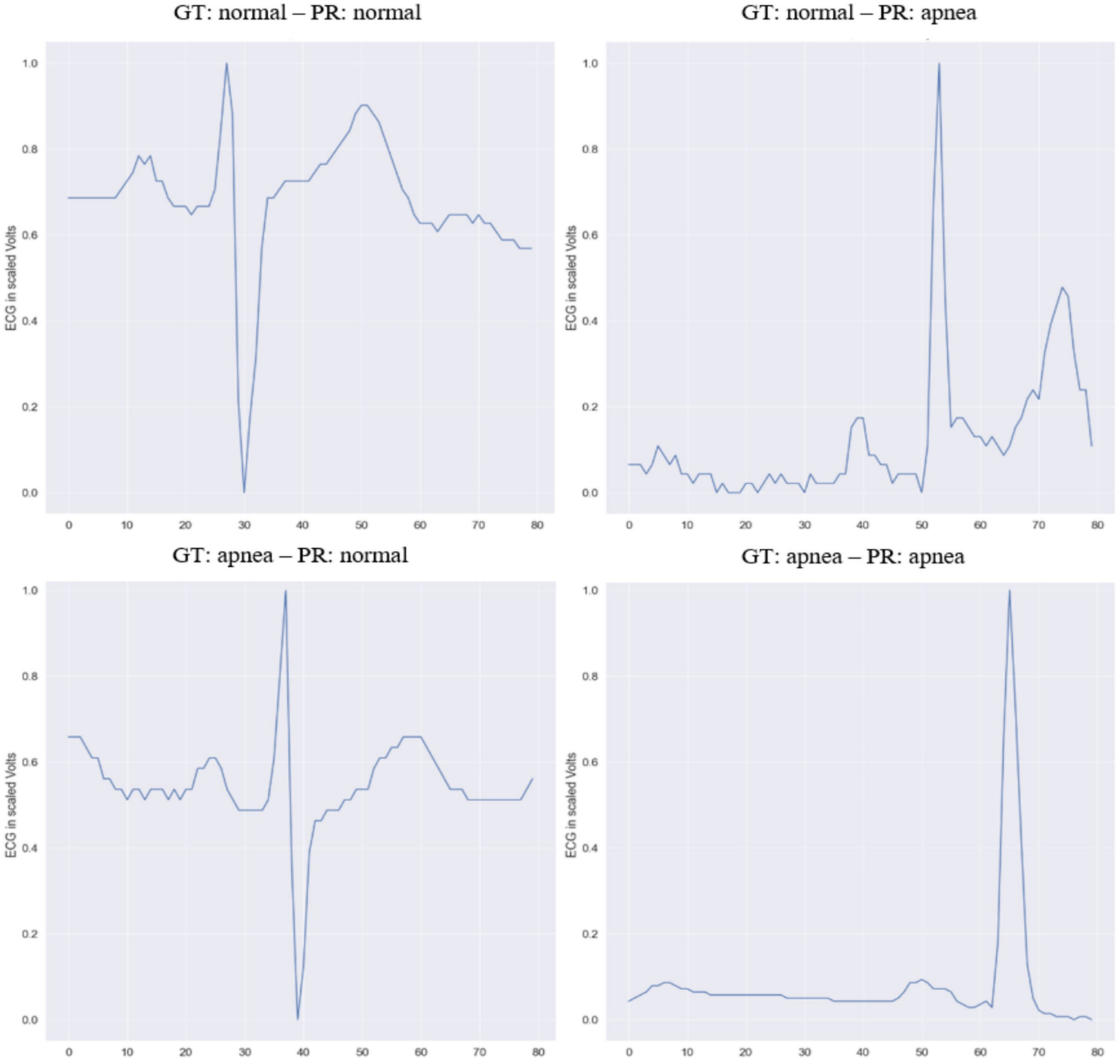
Predicted (PR) and ground truth (GT) events for 4 different samples from the testset, at 1-s intervals.

From the beginning, the proposed Transformer-based models showed performance that was comparable to, and sometimes better than, existing deep learning architectures for OSA classification. Adding the autoencoder helped the model capture temporal dependencies and represent the input data more effectively, improving its understanding of each sample’s position and context over time. However, In comparison with Bernardini et al. (2021), our model was tested on an independent test set. The AIOSA (Bernardini et al., 2021) results were obtained by dividing the dataset into separate training and validation sets, a practice that may introduce data leakage and consequently cast doubt on the generalizability of the reported outcomes (Goodfellow et al., 2016). Figure 7 shows the variable learning rate and loss for the first 18 epochs. Figure 8, showcases random samples from the test set for TP, TN, FP, and FP.

## 6 Conclusions and future work

The goal of this work is to develop an efficient tool to support clinical decision-making for OSA. To this end, we propose a Transformer-based deep learning framework for OSA detection using only ECG, capable of handling noisy waveforms without extensive preprocessing. We first applied the framework to OSASUD (Bernardini et al., 2022) dataset and proved that it outperforms other solutions. Then, we focused on trying different positional encoding, the performance of learnable positioned encoding was better than static positional encoding. In addition, we tried to speed up the training process using AdamW and weight decay.

Our proposed scheme, based on the Transformer encoder with a convolutional autoencoder, as a positioning encoding, that contains a convt layer, detects OSA events better than ResNet, LSTM, and CNN encoding (Bernardini et al., 2021); with F1 score equivalent to 0.863 and AUC-ROC equivalent to 0.852.

For future work, we plan to test the model on diverse datasets and incorporate additional PSG signals, such as thoracic effort (THO), abdominal effort (ABD), and EEG. Most current deep learning approaches detect apnea from a single lead, usually ECG, which offers limited clinical accuracy; as an expert physician remarked, “It would not be very accurate to depend solely on one lead. In practice, we look to different signals at the same time.” In addition, future work will include the collection of larger, multi-center datasets, external validation on independent cohorts, and comprehensive robustness analyses to strengthen model reliability and generalizability. We also plan to integrate Explainable AI techniques to provide clinicians with interpretable insights into model predictions. However, this is an expected direction for a new multi-disciplinary area, not to mention the expensive computational equipment that must be acquired to develop a decent DL model for multivariate time series data with a high sampling rate.

## Data Availability

The original contributions presented in the study are included in the article/supplementary material, further inquiries can be directed to the corresponding author.
